# Feasibility of Monitoring Response to Metastatic Prostate Cancer Treatment with a Methylation-Based Circulating Tumor DNA Approach

**DOI:** 10.3390/cancers16030482

**Published:** 2024-01-23

**Authors:** Thomas Büttner, Dimo Dietrich, Romina Zarbl, Niklas Klümper, Jörg Ellinger, Philipp Krausewitz, Manuel Ritter

**Affiliations:** 1 Department of Urology and Pediatric Urology, Venusberg-Campus 1, University Hospital Bonn, 53127 Bonn, Germany; niklas.kluemper@ukbonn.de (N.K.); joerg.ellinger@ukbonn.de (J.E.); philipp.krausewitz@ukbonn.de (P.K.); mritter@ukbonn.de (M.R.); 2 Department of Otorhinolaryngology, University Hospital Bonn, 53127 Bonn, Germany; dimo.dietrich@ukbonn.de (D.D.); romina.zarbl@ukbonn.de (R.Z.)

**Keywords:** metastatic prostate cancer, biomarkers, SHOX2, SEPT9, liquid biopsy

## Abstract

**Simple Summary:**

This study tackles the challenges of assessing treatment response in advanced prostate cancer. The standard test, measuring the PSA protein in blood, lacks reliability in some patients. We explored a method examining genetic material in blood samples. We focused on two methylation markers, *SHOX2* and *SEPT9*, as proxies of circulating tumor DNA. We collected blood samples from 11 patients with advanced prostate cancer undergoing different treatments. The results showed that all markers showed a response to treatment, especially *SHOX2*. This suggests that tracking advanced prostate cancer through liquid biopsy might harbor potential to monitor treatment effectiveness. This study is designed as a feasibility assessment and starting point, and more research with a larger group of patients is needed for confirmation.

**Abstract:**

Background: Metastatic prostate cancer (mPCA) poses challenges in treatment response assessment, particularly in cases where prostate-specific antigen (PSA) levels do not reliably indicate a response. Liquid biopsy, focusing on circulating cell-free DNA (ccfDNA) methylation analysis as a proxy for circulating tumor DNA, offers a non-invasive and cost-effective approach. This study explores the potential of two methylation markers, short stature homeobox 2 (SHOX2) and Septin 9 (SEPT9), as on-mPCA-treatment biomarkers. Methods: Plasma samples were collected from 11 mPCA patients undergoing various treatments. Quantitative assessment of hypermethylated SHOX2 (mSHOX2) and SEPT9 (mSEPT9) levels in ccfDNA was conducted through methylation-specific real-time PCR. Early and overall dynamics of PSA, mSHOX2, and mSEPT9 were analyzed. Statistical evaluation employed Wilcoxon tests. Results: mSHOX2 demonstrated a significant decline post-treatment in patients with a radiographic treatment response as well as in an early treatment setting. mSEPT9 and PSA exhibited non-significant declines. In individual cases, biomarker dynamics revealed unique patterns compared to PSA. Discussion: mSHOX2 and mSEPT9 exhibit dynamics on mPCA treatment. This proof-of-concept study lays the groundwork for further investigation into these markers as valuable additions to treatment response monitoring in mPCA. Further validation in larger cohorts is essential for establishing clinical utility.

## 1. Introduction

Numerous drug-based tumor therapy options are available for metastatic prostate cancer (mPCA) [1]. Among these, chemotherapy and radiopharmaceutical ligand treatment are particularly associated with adverse events [2,3]. To prevent unnecessary toxicity, a fast on-treatment response assessment is desirable. Normally, this evaluation is based on prostate-specific antigen (PSA) levels, whose response indicates a prolonged, progression-free and overall survival outcome [4,5,6,7,8]. However, in some cases PSA may potentially be an inadequate way to monitor disease [9]. mPCA can be PSA-negative or behave discordantly to the PSA trend due to inter- and intratumoral heterogeneity [10,11]. A post-hoc analysis of the ARCHES trial revealed that PSA progression and radiographic progression behaved inconsistently among a majority of mPCA patients treated with enzalutamide or a placebo, and in the CHAARTED trial, approximately 25% of patients with or without docetaxel showed progressive disease in imaging despite the absence of PSA progression [12,13]. These results emphasize the need for additional imaging throughout treatment and follow-up, as suggested in the European association of urology (EAU) 2023 guidelines [1]. Further biomarkers are therefore being investigated that may provide additional guidance, and similar to other tumor entities, liquid biopsy in mPCA has generated widespread interest [14]. Although certain blood-based tests exhibit promise in the context of diagnosis and therapy monitoring in metastatic prostate cancer, they rely on costly analysis of circulating tumor cells (CTCs) [15,16,17,18,19].

As an alternative, cost-effective non-invasive biomarkers, such as circulating cell-free DNA (ccfDNA), might hold the potential to monitor mPCA treatment. CcfDNA is released into the bloodstream as a consequence of tumor-related processes, which together with the presence of cancer-specific epigenetic changes, including aberrant methylation patterns, permits the detection of tumor-derived DNA [20]. Two extensively studied biomarkers, short stature homeobox 2 (SHOX2) and Septin 9 (SEPT9), have been investigated in various cancer types. As a result, hypermethylated SHOX2 (mSHOX2) and SEPT9 (mSEPT9) genes within ccfDNA have been identified as potent pan-cancer biomarkers and proxies for circulating tumor DNA (ctDNA) [21]. Further research has validated the diagnostic accuracy of mSHOX2 and mSEPT9, resulting in their endorsement as diagnostic biomarkers for lung and colorectal cancer with commercially available assay kits “Epi proColon” and “Epi proLung” [22,23].

The occurrence and diagnostic potential of mSHOX2 and mSEPT9 in prostate cancer remain areas of very limited investigation. Both markers exhibit dynamics during prostate biopsy and over the course of radical prostatectomy, indicating tumor cell release due to mechanical damage [24]. Additionally, promising data suggests the potential of mSEPT9 as a prognostic tool in mPCA [25].

This proof-of-concept study was undertaken with the aim of exploring the latent capabilities and dynamic attributes of quantitative SEPT9 and SHOX2 hypermethylation in liquid biopsy ccfDNA during mPCA pharmacological treatment, serving as on-treatment biomarkers. Clinically established assays were harnessed for the quantitative measurement of mSHOX2 and mSEPT9, with the objective of enhancing reproducibility and facilitating prospective clinical applications.

## 2. Materials and Methods

### Methods

With the approval of the local Ethics Committee (University Bonn, vote #348/19), we conducted a prospective collection of plasma samples from patients undergoing pharmacological treatment for mPCA. Eleven individuals with mPCA were included between September 2019 and July 2020. Patients underwent treatment with androgen deprivation therapy (ADT) with additional irradiation of the prostate according to the STAMPEDE trial (*n* = 1 [26]), docetaxel chemotherapy (*n* = 4), cabazitaxel chemotherapy (*n* = 4), a sequence of both chemotherapies (*n* = 1), or Lutetium-177 PSMA radionuclide therapy (*n* = 1). The standard of care for each treatment remained unaltered, including PSA tests and radiographic re-staging. The collection of blood samples for methylation marker analysis was contingent on the clinical availability of patients, with a maximum of 15 blood samples per patient.

The method of quantitative measurement of mSHOX2 and mSEPT9 has been described before [24]. In brief, blood was sampled into ethylenediaminetetraacetic acid (EDTA)-stabilized collection tubes, and processed within a 2 h timeframe. Probes were centrifuged to extract plasma, which was stored at −20 °C. DNA was isolated using the ammonium bisulfite method described by Jung et al. [27]. Then, quantification of mSEPT9 and mSHOX2 was carried out using SHOX2/SEPT9/ACTB triplex quantitative methylation-specific real-time PCR, as previously described in detail [27]. Actin-beta (ACTB) was employed as a reference standard and quantified to represent the total DNA content of the sample. The target regions for SEPT9 (chromosome 17:77,373,481e77,373,540) and SHOX2 (chromosome 3:158,103,550e158,103,661) were selected corresponding to the target sequences utilized in “Epi proColon” and “Epi proLung” as described before [27]. Both absolute and relative methylation levels were calculated using the ΔΔCT method, with relative methylation defined as the ratio of SEPT9/SHOX2 methylation to ACTB [28].

In order to analyze PSA, mSEPT9, and mSHOX2 on-treatment, we defined early treatment dynamics, referring to the biomarker changes in the first 20 days after treatment initiation. Only men with measurable PSA, mSEPT9, and mSHOX2 at baseline, as well as availability of the corresponding samples 10 and 20 days (+/−2) after starting therapy, were included but regardless of treatment response. Overall dynamics represent the development over the entire course of treatment in patients with a radiographically confirmed response. Response was defined as stable disease or partial/complete remission according to Response Evaluation Criteria In Solid Tumors (RECIST) criteria.

Statistical analysis was conducted using RStudio 2023.06.2 Build 561 (https://www.r-project.org/, accessed on 27 August 2023). Levels of mSEPT9 and mSHOX2 were reported as medians. For dynamic assessment, baseline values of mSHOX2, mSEPT9, and PSA were considered to be 100%, and further samples were compared in relation to the baseline using two-sided Wilcoxon rank sum tests. *p*-values less than 0.05 were considered statistically significant.

## 3. Results

Baseline characteristics of the cohort are summarized in Table 1. Most patients were treated in a castration-resistant mPCA (mCRPC) setting. PSA and methylation markers mSEPT9 and mSHOX2 presented a broad range of values at baseline. Radiographic treatment assessment was available in nine patients: two patients (18.2%) developed partial remission; stable disease occurred in five patients (45.5%) including a mixed response (see case presentation below). One patient (9.1%) developed progressive disease, and in three patients (27.3%) response was not available due to lost follow-up, switching of imaging technique, and rapid demise.

### 3.1. Early Treatment Dynamics

**Figure 1:**
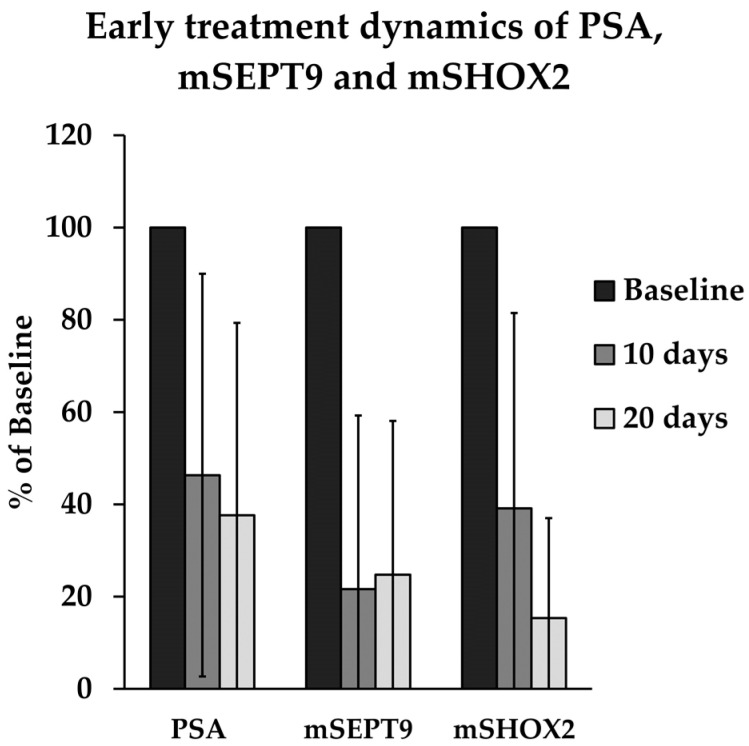
Early treatment dynamics of PSA, mSEPT9, and mSHOX2 in ccfDNA in six patients. Baseline measurement is given alongside mean relative amount in serial liquid biopsies 10 and 20 (+/−2) days after treatment initiation. All biomarkers exhibit a decrease, indicating treatment-associated dynamics. Differences from baseline to 20 days were significant for mSHOX2 (Wilcoxon *p* = 0.014), and revealed a non-significant trend for mSEPT9 and PSA (*p* = 0.126 and *p* = 0.306, respectively). Data are presented as means, with error bars indicating standard deviation. PSA = prostate-specific antigen, mSEPT9 = hypermethylated Septin 9, mSHOX2 = hypermethylated short stature homeobox 2.

Six patients met the criteria to be included in this analysis. All patients exhibited a decline in both PSA and methylation markers. Over the course of 20 days, mSEPT9 exhibited the fastest decline relative to the baseline, registering approximately one-quarter mean relative hypermethylation after 10 days (21.6%, interquartile range (IQR): 5.3–55.4%, standard deviation (SD) 37.6%). Nevertheless, no further reduction was observed in the subsequent weeks (20 days: 24.8%, IQR: 4.0–47.6%, SD: 38.5%). PSA and mSHOX2 showed a more gradual but consistent decline, reaching 37.6% of baseline for PSA (IQR: 5–52.6%, SD: 41.7%) and 15.3% for mSHOX2 (IQR: 3.2–18.4, SD: 21.7%) after 20 days of therapy. Differences from baseline to 20 days revealed statistical significance for mSHOX2 (Wilcoxon *p* = 0.014), with a non-significant trend for PSA and mSEPT9 (*p* = 0.306 and 0.126, respectively). Early dynamics are elucidated in Figure 1.

### 3.2. Overall Dynamics

**Figure 2:**
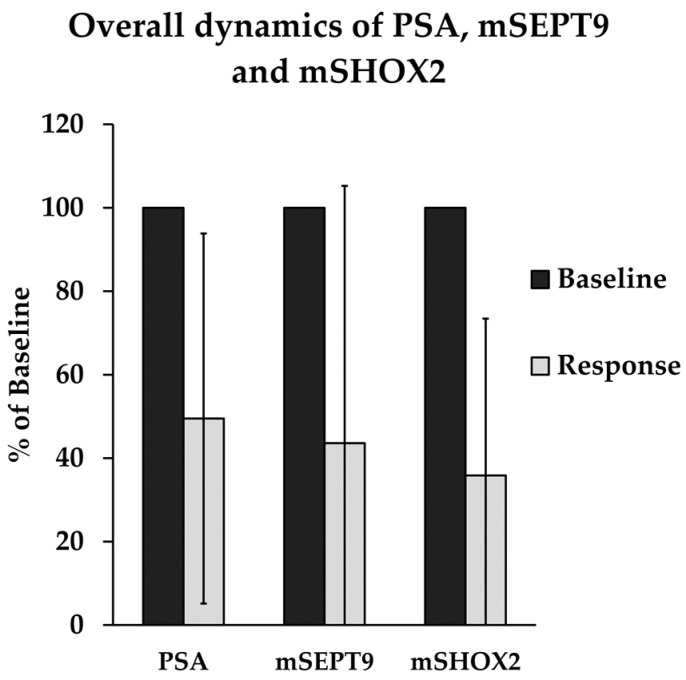
Overall dynamics of PSA, mSEPT9, and mSHOX2 in ccfDNA in seven patients. Baseline measurement is given alongside mean relative amount after radiographically confirmed treatment response. All biomarkers show a decline, suggesting treatment-associated dynamics. Differences were significant for mSHOX2 (Wilcoxon *p* = 0.028), with a non-significant trend for mSEPT9 and PSA (*p* = 0.263 and *p* = 0.240, respectively). Data are presented as means, with error bars indicating standard deviation. PSA = prostate-specific antigen, mSEPT9 = hypermethylated Septin 9, mSHOX2 = hypermethylated short stature homeobox 2.

Seven patients could be included in this analysis. All biomarkers (PSA, mSEPT9, mSHOX2) showed a decline in this two-point measurement. mSHOX2 reached the lowest mean value with 36.3% of baseline (IQR: 7.2–50.7%, SD 37.5%), followed by mSEPT9 with 43.6% (IQR 0.9–82.7%, SD 61.7%) and PSA with 49.5% (IQR 3.3–87.5%, SD 44.3%). Comparing the baseline to the measurement subsequent to treatment, the mSHOX2 decrease reached statistical significance, denoted by a *p*-value of 0.028. Meanwhile, PSA and mSEPT9 differences remained insignificant, indicating a possible trend (*p* = 0.240 and 0.263, respectively). Overall dynamics are illustrated in Figure 2.

### 3.3. Case Study

**Figure 3:**
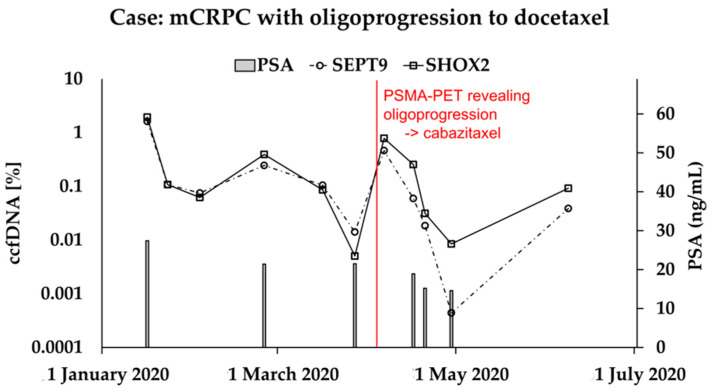
Case presentation of serial measurements of PSA, mSEPT9, and mSHOX2 in ccfDNA in a mCRPC patient. Interim staging after three cycles of docetaxel revealed a radiographic mixed response (lymph node metastasis oligoprogression) resulting in a switch to cabazitaxel, eventually leading to stable disease. While PSA showed little dynamics, both methylation markers congruently give an indication of tumor progression matching the oligoprogression of the disease depicted on PSMA-PET/CT. PSA = prostate-specific antigen, mSEPT9 = hypermethylated Septin 9, ccfDNA = circulating cell-free DNA, mSHOX2 = hypermethylated short stature homeobox 2, mCRPC = metastatic castration-resistant prostate cancer, PSMA-PET/CT = prostate-specific membrane antigen positron emission topography and computed tomography.

A consistent correlation between PSA and methylation patterns was noted in the majority of patients; however, we wish to highlight one case in particular: A 76-year-old male patient underwent docetaxel treatment subsequent to the progression of metastatic disease involving osseous and lymphogenous sites while on enzalutamide. Following three cycles, scheduled staging (prostate-specific membrane antigen positron emission topography and computed tomography; PSMA-PET/CT) demonstrated a favorable response in the osseous metastases, yet indicated asymptomatic progression in the pelvic lymph node metastases. Consequently, the patient underwent a transition to cabazitaxel, yielding a state of stable disease in the subsequent staging evaluation. The development of the biomarkers PSA, mSHOX2, and mSEPT9 is shown in Figure 3. Notably, PSA displays minimal dynamics, specifically demonstrating no progression throughout the entire treatment regimen. In contrast, the methylation markers distinctly reveal secondary progression to docetaxel and subsequent response to cabazitaxel.

## 4. Discussion

Liquid biopsy, specifically ccfDNA methylation analysis, demonstrates a shift towards non-invasive and cost-effective monitoring tools. The exploration of mSHOX2 and mSEPT9 as potential on-treatment biomarkers builds on their established roles in other cancer entities, underlining the importance of investigating their diagnostic and prognostic potential in the context of mPCA. The evaluation of treatment response in mPCA poses a multifaceted challenge, given the limitations inherent in PSA-based response assessments [9,10]. A substantial amount of mCRPC patients do not respond to chemotherapy or radioligand therapy [29,30,31]. In light of the potentially severe side effects, early identification of men without treatment response is desirable. As evidenced in our case illustration and corroborated by findings from the CHAARTED and ARCHES trials, the PSA value may not consistently indicate progression [12,13]. In this context, ccfDNA methylation markers mSHOX2 and mSEPT9 show promise for on-treatment monitoring. Its utilization could serve as a cost-effective adjunct to PSA in guiding decision making in the event of a discrepancy between imaging and PSA value development. Furthermore, they could function as markers to prompt delayed or early imaging, particularly considering the absence of established guidelines defining imaging intervals in mPCA [1]. In the rare scenario of “PSA-negative” metastatic disease, mSHOX2 and mSEPT9 may play a role, mitigating the absence of reliable non-invasive surrogate markers for tumor burden that often lead to diagnostic delays and hinder effective management [11].

With our findings, a rationale for investigating these methylation markers as biomarkers for medical anti-tumor therapy for mPCA emerges. While our study represents an initial feasibility analysis not yet powered to detect significant deviations during treatment, our data provide valuable insights: mSEPT9 and mSHOX2, similar to PSA, show dynamics during the course of therapy, which can even reveal significant differences in small cohorts for mSHOX2. This suggests a possible correlation to tumor burden and metabolic activity as shown before [24]. Additionally, our study aligns with existing evidence, affirming the value of quantitative analysis of mSEPT9 and mSHOX2 in plasma for individually monitoring the response of mPCA to chemotherapy [21]. Looking beyond response prediction, survival analyses of CTC dynamics during mPCA treatment confirmed their prognostic value [18,19]. Further research may investigate whether the surrogates mSEPT9 and mSHOX2 can provide similar prognostic information, offering a more cost-efficient alternative.

However, in addition to reliability, responsiveness of biomarkers plays an important role in the on-treatment setting. Particularly in mCRPC, it takes up to four cycles before a significant survival benefit becomes apparent from PSA half-life [32]. Rapid-response markers would be desirable to shorten this period during the ongoing burden of treatment side effects. The initial dynamics observed in this study regarding mSEPT9 and mSHOX2 imply the potential for discernible changes within a three-week timeframe after initiating therapy.

The case study offers a compelling illustration of the potential clinical utility of these biomarkers. The methylation markers’ behavior in response to different chemotherapy agents emphasizes the complementary role of liquid biopsy in capturing treatment-specific nuances and guiding therapeutic decisions to overcome the divergence of PSA and imaging revealed in large-scale trials [12,13]. Standardized recommendations on the intervals of imaging in mCRPC are pending. The EAU guideline currently does not contain a recommendation; the Prostate Cancer Radiographic Assessments for Detection of Advanced Recurrence (RADAR) group recommends imaging only at the end of treatment in the absence of symptoms and clinical progression [1,33]. However, based on these suggestions, the diagnosis of oligoprogression might have been significantly delayed in the presented case. Additive low-cost biomarkers such as mSEPT9 and mSHOX2 can be particularly valuable here to aid clinicians’ decisions to initiate imaging, allowing for a timely detection of progression.

This study’s commendable strengths are evident in the strict adherence of its methods to established protocols [27]. Quantitative methylation-specific real-time PCR for assessing mSHOX2 and mSEPT9 ensures robust and reproducible measurements, critical for future clinical applications. Moreover, mSHOX2/mSEPT9 analysis can be conducted using commercially available assays (“Epi proColon” and “Epi proLung”) targeting the corresponding DNA sequences.

Despite the gained insights, our study has certain limitations. First, due to a real-world treatment setting, both serial liquid biopsies for early dynamics and radiographic response were not collected at certain times. As a result, not all patients can be included in the final analyses, which compromises the conclusions. Nonetheless, encouraging results support further investigation in a more controlled trial setting. Second, ccfDNA is subject to limitations, with highly variable individual baseline levels reflecting unique tumor biology, including some patients with a negative mSEPT9 baseline. This creates a heterogeneous pattern of relative hypermethylation. Thus, comparable to PSA, the biomarkers appear to be useful only in the dynamic response rather than in a single-point measurement. Third, designed as a pilot feasibility report, it does not allow for sound conclusions to be drawn about the dynamics being monitored. Nevertheless, this proof-of-concept study lays the groundwork for the exploration of mSHOX2 and mSEPT9 as on-treatment biomarkers in mPCA, especially in “PSA-negative” or incongruent PSA level cases. Our findings contribute to the growing body of evidence supporting the use of liquid biopsy for monitoring metastatic prostate cancer treatment. Further validation studies with larger cohorts are warranted to establish the clinical utility and generalizability of mSEPT9 and mSHOX2 in clinical practice.

## 5. Conclusions

Additional liquid biopsy-based biomarkers are desirable to overcome the shortcomings of PSA in treatment monitoring of mPCA, and pan-cancer ccfDNA methylation markers mSEPT9 and mSHOX2 may provide a promising tool. However, to date, limited data is available for them in mPCA. In this proof-of-concept study, we explored the dynamics of mSHOX2 and mSEPT9 as on-treatment biomarkers in mPCA. Encouragingly, both mSHOX2 and mSEPT9 demonstrated relative declines during therapy, aligning with PSA dynamics, and captured treatment-specific nuances in individual cases. Despite limitations, our study supports further investigation, emphasizing the complementary role of liquid biopsy in monitoring mPCA treatment and opening up the prospect of a cost-effective surrogate for neoplastic load.

## Figures and Tables

**Table 1:** Baseline characteristics.

Characteristic	*n* = 11
Age	
Median (IQR)	70.6 (67.4–76.5)
Range	59.7, 81.3
ECOG	
0	6 (54.6%)
1	4 (36.4%)
2	1 (9.1%)
Castration-resistant	
Yes	9 (81.2%)
No	2 (18.8%)
PSA (ng/mL)	
Median (IQR)	52.8 (27–85.5)
Range	3.0–2193
mSEPT9 in ccfDNA (%)	
Median (IQR)	0.72 (0.49–5.81)
Range	0, 22.3
mSHOX2 in ccfDNA (%)	
Median (IQR)	0.50 (0.08–1.94)
Range	0.1, 8.3

IQR = Interquartile Range, ECOG = Eastern Cooperative Oncology Group Performance Status Scale, PSA = prostate-specific antigen, mSEPT9 = hypermethylated Septin 9, ccfDNA = circulating cell-free DNA, mSHOX2 = hypermethylated short stature homeobox 2.

## Data Availability

The raw data supporting the conclusions of this article will be made available by the corresponding author on request.
